# Continuous Blown Film Preparation of High Starch Content Composite Films with High Ultraviolet Aging Resistance and Excellent Mechanical Properties

**DOI:** 10.3390/polym13213813

**Published:** 2021-11-04

**Authors:** Bowen Lin, Chengqiang Li, Fangping Chen, Changsheng Liu

**Affiliations:** 1 Engineering Research Center for Biomedical Materials of Ministry of Education, East China University of Science and Technology, Shanghai 200237, China; bowen_lin_a@163.com (B.L.); chengqiang_li@163.com (C.L.); 2 Key Laboratory for Ultrafine Materials of Ministry of Education, School of Materials Science and Engineering, East China University of Science and Technology, Shanghai 200237, China

**Keywords:** lignin, film, PBAT, high starch content, ultraviolet aging resistance

## Abstract

Starch/PBAT blown films with high ultraviolet aging resistance and excellent mechanical properties were prepared by introducing lignin with polyurethane prepolymer (PUP) as a starch modifier and physical compatibilizer and 4,4′–methylene diphenyl diisocyanate (MDI) as a crosslinker. Starch was modified by reacting the NCO groups of the PUP with the OH groups of the starch to form a carbamate bond. The mechanical properties, hydrophobic properties, ultraviolet barrier, ultraviolet aging properties and microscopic morphology of starch/PBAT films with different contents of lignin were investigated. The results showed that the starch/PBAT films were blown continuously. The addition of lignin did not decrease the mechanical properties. On the contrary, the film with 1% lignin possessed the excellent mechanical properties with longitudinal tensile strength of 15.87 MPa and the elongation at a break of 602.21%. In addition, the higher the lignin content, the better the UV blocking effect. The introduction of lignin did not affect the crystalline properties but improved the hydrophilic properties and sealing strength of the high starch content composite films.

## 1. Introduction

Plastics are widely used in packaging, isolation and mulch due to their low cost, high mechanical properties and easy processing characteristics [1]. Mulch films play an important role in inhibiting the growth of weeds, improving water use efficiency and increasing crop yields in modern agriculture [2]. However, conventional mulch films, mainly composed of polyethylene or polyvinyl chloride, are non-degradable and derived from non–renewable petroleum. Their excessive usage may result in the accumulation of solid waste and deteriorating environment seriously [3,4]. After crops are harvested, a large amount of residual film remains in the soil, which leads to a decrease in soil moisture and nutrient permeability and is not conducive to crops [5]. Under the promotion of national policies, the emergence of biodegradable mulch will effectively solve the problem of residual pollution of non-degradable mulch.

Starch is not only an abundant resource of low cost with high renewability and biodegradability, but its degradation products also facilitate the growth of crops without the risk of compacted soil, indicating that films based on starch are suitable for agricultural mulch. However, pure starch cannot be blown into films due to the lack of thermoplastic properties and processability. Blending with degradable polyesters such as poly (butylene adipate co-terephthalate) (PBAT) and poly(lactic acid) (PLA) is a promising alternative to overcome the limitation of starch and lower the cost of pure polyesters to some extent.

Although the composites films were prepared by blending starch and degradable polyester by many researchers, the starch content in the composite films is relatively low (usually less than 30%). The higher the starch content, the lower the cost of the composite but the higher the processing difficulty. More importantly, with the increase of starch content, the starch and polyester tend to be difficult to mix evenly because starch is hydrophilic while polyesters are hydrophobic. In addition, there is obvious phase separation between starch and degradable polyester. The mechanical properties of composite films will decrease rapidly at the high content of starch. As a result, the agricultural mulch films on the current market are mainly PE non-degradable mulch films or low starch content composite degradable films.

Low cost and high mechanical properties are the goal of biodegradable agricultural mulch films. Improving the starch content of the composite films, enhancing the interface bonding between starch and polyesters and continuous preparation play a vital role in the development of mulch films. PBAT is one of the degradable polyesters prepared by copolymerization reaction between butylene adipate and butylene terephthalate. It possesses excellent ductility, high elongation at break, high thermal stability and effective resistance to external impact. Therefore, in this study starch/PBAT composite materials with high strength and high starch content were attempted to prepare by in–situ reaction and melt blending with 4,4′–methylene diphenyl diisocyanate (MDI) as a bifunctional crosslinking agent and polyurethane prepolymer (PUP) as a starch modifier and physical compatibilizer.

In addition, the biodegradable mulch films are directly exposed to sunlight during use. Ultraviolet light will accelerate the aging of the film, resulting in performance degradation and a shorter life cycle. Therefore, it is meaningful and necessary to study anti-ultraviolet aging biodegradable film, aiming to extend the life cycle of the film and slow down its photodegradation.

The evidence for the abundant reserves of lignin is that it can be extracted from wood, can be obtained from some plants and agricultural products, and it is also a biodegradable and sustainable material [6,7,8,9,10,11,12]. Its utilization as an environmentally friendly biodegradable material and as an alternative to petroleum-based materials has also attracted attention [13]. Lignin is the second-largest renewable and biodegradable natural resource on earth (after cellulose), and it is a natural resource-based on aromatic units [14]. In addition to low cost, lignin also has many advantages, such as high carbon content, biodegradability, antioxidant and antibacterial activities. However, about 50 million tons of lignin are produced each year, but less than 2% is used in high-value products, and the rest is only used as a waste treatment or used in low-value applications, such as heating and power generation [15]. Lignin has high impact strength and good heat resistance and can also improve the fluidity and processing properties of the thermoplastic polymer matrix. In addition, it is light in weight and low in cost, so the cost and weight of composite materials can be reduced. It shows great commercial potential and can be used as a reinforcing filler for the development of green and biodegradable materials. In addition, lignin is also a natural substance with anti-oxidation and anti-ultraviolet aging. Sadeghifar et al. prepared a cellulose-lignin film. It is reported that cellulose film containing 2% lignin showed around 100% absorption of UV-B (280–320 nm) and more than 90% of UV-A (320–400 nm) [16]. This indicates that lignin provided high-efficiency UV absorption properties for the film. However, it is also reported that the addition of lignin reduced the mechanical properties of cellulose films. Xing et al. introduced lignin into PBAT and successfully prepared a biodegradable mulch film with ultraviolet barrier and anti-ultraviolet aging functions [17]. Xing et al. introduced lignin into PBAT and successfully prepared a biodegradable mulch film with ultraviolet barrier and anti-ultraviolet aging functions [17].

In this study, it was attempted to introduce lignin into starch–based composite films, aiming to endow the starch–based films with UV aging resistance. In addition, polyurethane prepolymer (PUP) was used to modify starch, and a small amount of crosslinking agent MDI was added to the system to improve the mechanical properties of the film. Starch–based composite films with different lignin contents are prepared by melt blending and extrusion blown methods. The effects of lignin with different contents on the tensile properties, crystallization properties, heat sealing properties, hydrophobic properties and UV resistance properties of the starch/PBAT composite film were also investigated.

## 2. Materials and Methods

### 2.1. Materials

Corn starch was purchased from Nanjing Ganzhiyuan Sugar Industry Co., Ltd. (Nanjing, China). The lignin was purchased from Shanghai Xianding Biological Technology Co., Ltd. (Shanghai, China). 4,4′-methylene diphenyl diisocyanate (MDI) and Polybutylene succinate diol were purchased from Aladdin Reagent Co., Ltd. (St. Louis, MO, USA). Poly (butylene adipate–*co*–terephthalate) (Ecoflex C1200) were purchased from BASF (Ludwigshafen, Germany).

### 2.2. Preparation of Modified Starch

Modified starch was prepared by polyurethane prepolymer (PUP) and corn starch, according to our previous work, with a little modification [18]. In short, starch and PUP were dried at 70 °C for 12 h. Starch and PUP with a mass ratio of 3:1 were reacted at 90 °C for 30 min in an open-close internal mixer (CF-1LKH, Guangdong, China) at a rotation speed of 30 rpm. The modified starch was then put into an oven for curing at 100 °C for 12 h. Finally, the modified starch was cooled to room temperature, crushed with a pulverizer, and stored for the following experiment.

### 2.3. Preparation of Anti-Ultraviolet Aging Starch-Based Film

The anti-ultraviolet aging starch-based composites were prepared by reacting modified starch, PBAT, MDI and lignin in an internal mixer at 180 °C for 30 min, as listed in Table 1. A single-screw extrusion blown film machine was used to prepare starch-based composite films. The diameter of the die of the blown film auxiliary machine is 30 mm, and the interval between the discharge ports is 10 mm. The temperature of the film blowing machine from the feeding area to the die is set to 130 °C, 135 °C, 140 °C, 145 °C, and 150 °C in sequence. The feed rate was set to 20 rpm, and the single screw conveying rate was set to 30 rpm.

### 2.4. Methods

#### 2.4.1. Infrared Spectroscopy Analysis

To show whether the addition of lignin to the composite material will affect the structure of the composite material, put each group of starch-based film in a Fourier infrared spectrometer (Thermo Electron Corporation, Carlsbad, CA, USA) for infrared test. The infrared wavelength range is set to 4000–400 cm^−1^. After the test results come out, the peaks appearing in the obtained infrared spectrum are compared with the standard peak spectrum to determine the concentration of each typical group in the film sample to analyze the degree of reaction of each component.

#### 2.4.2. Crystal Morphology Analysis

X–ray diffractometer (D8 Advance, Japan) was used to study the crystalline morphology of starch-based films doped with different lignin content. The test parameters are as follows: the test voltage and current intensities were 40 kV and 100 mA, the scanning speed was 3°/min and the scanning range was 5–75°.

#### 2.4.3. Contact Angle and Water Vapor Transmission Rate

The contact angle of starch-based films doped with different lignin content was tested on a contact angle measuring instrument (JC2000D2, Zhongchen Digital Technology Equipment Co., Ltd., Shanghai, China). Cut the film into a size of 3 × 3 cm and place it on the test platform for testing. Wait until the water drops on the surface of the film. After waiting for 10 s, freeze the image and read the degree. Test multiple times and take the average value.

To compare the water vapor transmission rate of the starch-based film before doping with lignin, it was tested according to the ASTM E96-00 test standard. The test process is as follows: Take several centrifuge tubes with a diameter of 27 mm and introduce about 2/3 of the volume of purified water into the centrifuge tubes. The mouth of the tube was sealed with the sample film of each group, and the total mass of the centrifuge tube and the sample film was weighed and recorded as $\;M_{0}$. In addition, the control group was an unsealed centrifuge tube filled with an equal amount of purified water. Place all the samples in an 85% humidity environment (saturated ammonium sulfate solution) and a constant temperature box at 37 °C. After 24 h, samples will be weighed again and recorded as $\;M_{1}$. Five parallel samples are set for each group. The water vapor transmission rate (*WVTR*) was calculated according to Formula (1):

(1) $$WVTR=\frac{\left(M_{1}-M_{0}\right)}{D\times A}\times 100\%$$

*D* (day) is the time in the incubator; *A* (m^2^) is the area of the centrifuge tube.

#### 2.4.4. Microscopic Morphology Observation

The microscopic morphology of starch-based films with different lignin contents was observed by scanning electron microscope (S4800, Hitachi Limited, Tokyo, Japan). After spraying gold for 60 s, the surface morphology of the film was observed under 15 kV acceleration voltage.

#### 2.4.5. Mechanical Performance Characterization

According to the GB/T 1040.3-2006 test standard, the mechanical properties of starch–based films with different lignin contents are tested using a universal testing machine (CMT2503). The film is cut into dumbbell-shaped splines with a narrow neck width of 4 mm and a gauge length of 25 mm. The stretching rate is 100 mm/min. For each type of film, at least five samples are tested.

#### 2.4.6. Ultraviolet-Visible Light Absorption Performance Test

To explore the influence of lignin on the optical properties of the starch/PBAT composite film, UV-VIS spectroscopy was performed on each film with an ultraviolet-visible spectrophotometer (UV-2600, Shimadzu, Kyoto, Japan). First, preheat the machine for half an hour to ensure the stability of the light source. Test the absorption of different light waves in the 200–800 nm wavelength range of each group of films.

#### 2.4.7. UV Aging Performance Test

According to the ASTM G154-06 test standard, each group of the starch-based film is subjected to an ultraviolet aging test in an ultraviolet aging box. The UV aging conditions are set as follows: the constant temperature is set to 60 °C, and the UV light is continuously irradiated for 100 h. After the aging is over, the mechanical properties of the film are tested. The preservation rate (CR, the ratio of the mechanical properties after UV aging to the ratio before aging) is calculated to determine the anti-UV aging effect of the film incorporated with lignin.

#### 2.4.8. Heat Sealing Performance Test

According to the test method of QB/T2358-98 standard, the starch-based films with different lignin contents are cut into strips of 75 mm × 15 mm. Align the two films and place them on the thermoplastic machine, and heat for 10 s with a sealing width of 2 mm. After cooling to room temperature, the mechanical properties are conducted on a universal testing machine at the vertical stretching speed of 300 mm/min. The distance between the clamps is set to 50 mm and the maximum load during the peeling process of the two films are recorded. The maximum load is the sealing strength of the film, in N/15 mm. Test multiple times and take the average value.

## 3. Results and Discussion

### 3.1. Infrared Results of Composite Materials with Lignin

**Figure 1:**
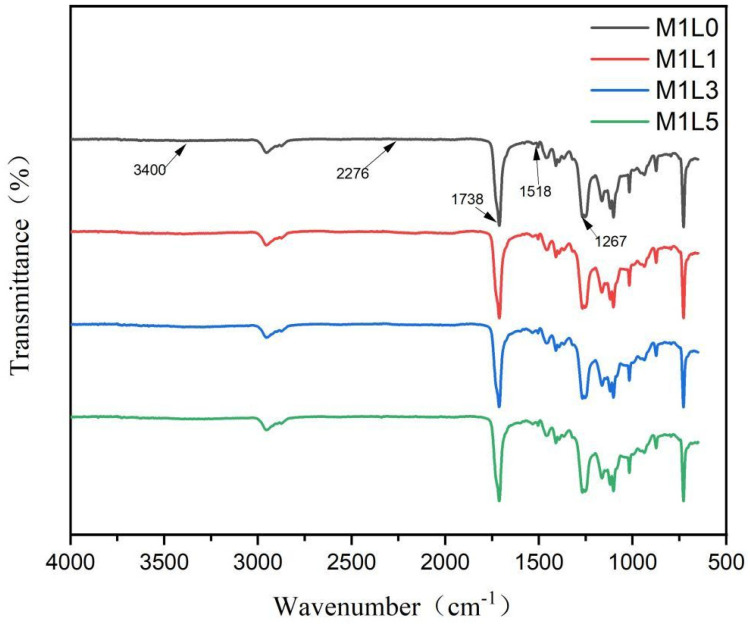
FTIR spectra of starch/PBAT films with different lignin contents.

The figure shows the FTIR spectra of starch/PBAT composite film doped with different content of lignin. As shown in Figure 1, the four groups of starch/PBAT composite materials all have an absorption peak at 1518 cm^−1^, which is attributed to the presence of N-H in the urethane bond of the urethane [19]. Moreover, the absorption peaks at 1738 cm^−1^ and 1267 cm^−1^ are due to the stretching vibration of the C–O and C=O group pairs in the urethane [19,20]. In addition, previous literatures reported that a broad vibration absorption peak near 3400 cm^−1^ was assigned to the hydroxyl groups in starch and an absorption peak at 2276 cm^−1^ was ascribed to the isocyanate groups in MDI [19,21,22,23]. However, none was found near 3400 cm^−1^ or 2276 cm^−1^ in the FTIR spectra of starch/PBAT composite film in our study. It indicated that the –OH groups of starch and the –NCO groups in the crosslinking agent were nearly fully reacted. Furthermore, Figure 1 showed that the addition of lignin did not affect the infrared spectra and the structure of composite films.

### 3.2. Crystal Structure of Starch-Based Films

**Figure 2:**
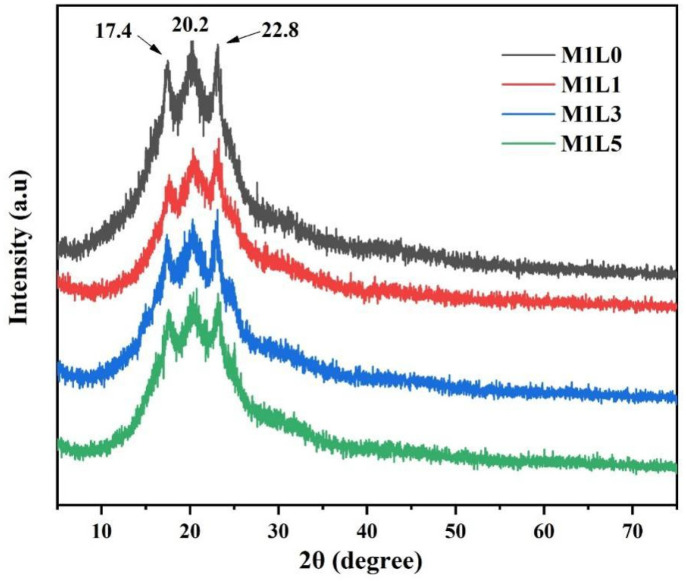
X-ray diffraction patterns of starch/PBAT films with different lignin contents.

The XRD curves of starch–based films with different lignin content were shown in Figure 2. The main diffraction peaks in the curve were 17.4°, 20.2° and 22.8°. Among them, 22.8° represented the typical type A crystal of starch [24]. The five characteristic diffraction peaks of PBAT were displayed at 16.3°, 17.4°, 20.2°, 23.1° and 25.0°, respectively, corresponding to the planes of (011), (010), (110), (100) and (111), respectively, which represented the α form of PBAT triclinic crystal accumulation [25]. Therefore, the diffraction peaks around 17.4° and 20.2° were due to the introduction of semi–crystalline PBAT. The lignin was a kind of non-crystalline three-dimensional network phenolic polymer. Lignin did not show any XRD diffraction peaks due to its amorphous state [26]. Therefore, the four groups of films only showed the crystalline structure of starch and PBAT, and the addition of lignin did not cause the appearance of new crystalline peaks. However, as the lignin content increased, it was obvious that the intensity of the diffraction peaks decreased slightly. This may be caused by the increase of heterogeneous nucleation points and the molecular interaction between lignin and starch.

### 3.3. Hydrophilic and Hydrophobic Properties

**Figure 3:**
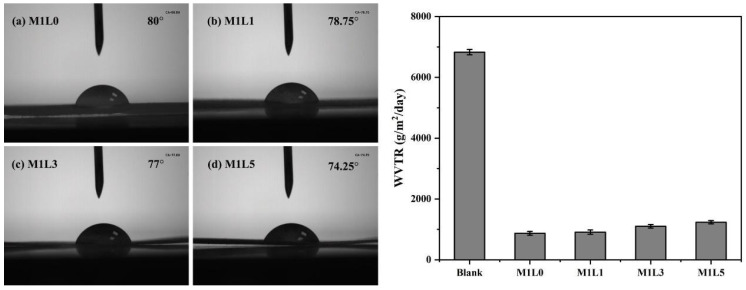
Contact angle of the composite film (left) and WVTR of the composite film (right) of starch/PBAT films with different Lignin contents: (**a**) M1L0, (**b**) M1L1, (**c**) M1L3, (**d**) M1L5.

The contact angle and water vapor transmission rate performance of starch/PBAT films with different lignin contents were displayed in Figure 3. and Table 2. The blank group is the samples without MDI and lignin. The characterization of water vapor transmission rate is based on the ASTM E96-00 test standard. The hydrophilic and hydrophobic properties of the film depended on the hydrophilic groups of the composite material. Figure 3 showed that the contact angle of M1L0 and M1L5 were 80° and 74.25°, respectively. It could be seen that the addition of lignin decreased the contact angle of the starch–based films to a certain extent. And the higher the lignin content, the more obvious the decrease. This was mainly due to the surface of lignin with a large number of hydrophilic hydroxyl and carboxyl groups.

Compared with the blank group, four groups of starch/PBAT films with different lignin contents films all had the obvious effect of the water vapor barrier. However, as the content of lignin increased, the water vapor transmission rate of the film was on the rise which meant that the water vapor barrier performance of the film decreased. Because lignin contains a large number of hydrophilic groups, its water absorption is extremely strong. When water molecules pass through the composite film, the free volume of molecular chains in the film increases, resulting in larger gaps between molecular chains and making it easy for water vapor to pass. Therefore, the introduction of lignin with a certain amount is favorable for improving the water vapor barrier performance of the starch-based composite films.

### 3.4. Morphology Observation

**Figure 4:**
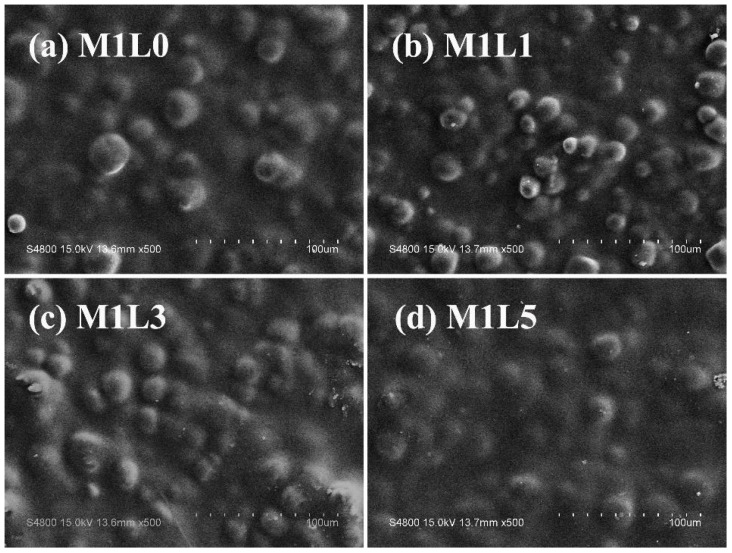
Scanning electron microscopy images of the surface morphologies of starch/PBAT films with different lignin contents under 500 times magnification: (**a**) M1L0, (**b**) M1L1, (**c**) M1L3, (**d**) M1L5.

The microscopic surface morphology of starch-based films with different lignin content was shown in Figure 4. Since the polyurethane prepolymer (PUP) reacted with the hydroxyl groups on the starch to form a urethane bond, the starch was chemically modified to improve the hydrophobicity. A polyurethane layer with good compatibility with PBAT was formed on the surface of starch granules, which can be supported by the small number of smooth particles observed on the surface of the sample [18]. It can be seen from Figure 4 that under the dual effects of PUP and MDI, starch and PBAT have good compatibility without significant phase separation. In addition, modified starch was distributed in the PBAT. Lignin was dispersed in the starch/PBAT system, which was attributed to the shear dispersion effect of the internal mixer. The results showed that the addition of lignin had no agglomeration phenomenon, and the starch–based film system still had good compatibility, which provided a guarantee for the maintenance of mechanical properties.

### 3.5. Mechanical Properties

**Figure 5:**
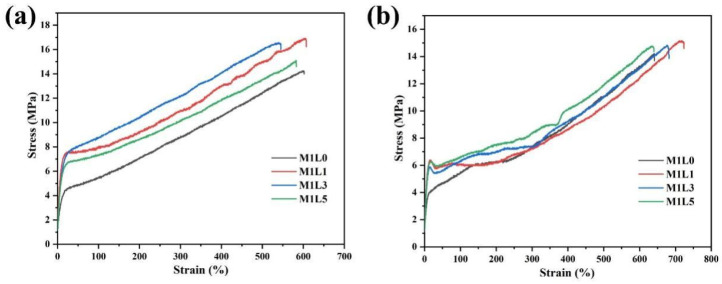
Tensile stress–strain curves of starch films with different lignin contents (**a**) longitudinal stretching and (**b**) horizontal stretching.

The stress–strain curves of starch/PBAT films with different lignin content were shown in Figure 5. Due to the influence of the film blowing ratio and traction ratio in the film blowing process, the mechanical properties of the film in different directions were different, and the longitudinal film performance (Figure 5a) was often higher than the transverse performance (Figure 5b). The specific longitudinal and transverse performance parameters of starch-based films with different lignin content were listed in Table 3. in detail. It can be seen from the results that the addition of lignin did not adversely affect the starch film. On the contrary, the addition of lignin effectively improved the tensile properties of the film. With the increase of lignin content, the strength initially increased and then decreased. The elongation at break also increased after the addition of lignin. Within the partial confidence interval, the elongation at break appears to overlap in value. This may be caused by the incomplete distribution of lignin in the structure. When the lignin content was 1 wt.%, the longitudinal tensile strength of the film was 15.87 MPa and the transverse tensile strength was 15.31 MPa, and its tensile performance was significantly higher than that of the sample without lignin. It was reported in the literature that lignin with an amorphous 3D structure and an aromatic backbone, when blended with a thermoplastic polymer, can generally enhance its strength or stiffness, while its ductility was significantly reduced [14]. The results of this experiment were consistent with the literature It can be seen from the stress-strain curve that the addition of lignin significantly increased the starting point of stress yield, and increased the hardness and elastic modulus of the film. There was also a downward trend in elongation at break. When the lignin content was 1 wt.%, the improvement of the film performance was attributed to the strong rigidity of the lignin itself and the good resistance to deformation. In addition, due to the low content of lignin, it can be dispersed more uniformly in the system. As the lignin content continued to increase, the toughness of the film decreased slightly. This may be caused by uneven lignin dispersion, causing the film to break at uneven places. In addition, combining the four sets of film data, it could be found that the mechanical properties of starch-based films were significantly improved due to the physical and chemical double compatibilization effects of MDI crosslinking agent and PUP.

In addition, it can be seen from Figure 4 and Table 3 that the longitudinal stretch ability of the film was higher than that of the transverse direction, while the elongation at break of the transverse direction of the film was better than that of the longitudinal direction. This was mainly related to the blow–up ratio and traction speed when the film was blown. This means that the arrangement of molecules in the horizontal and vertical directions will be different during the film forming process. In general, the molecular chains of the film have a higher degree of orientation in the longitudinal direction, which promotes stronger mechanical properties. By controlling the appropriate inflation ratio, the transverse and longitudinal properties of the film can be very close. However, the blow–up ratio of the film should not be too large. Otherwise, it will easily lead to instability of the film bubble and a significant drop in performance. The high elongation at break in the transverse direction may be attributed to the fact that the molecular segments were not well oriented during the film blowing process.

### 3.6. Ultraviolet-Visible Light Absorption Analysis

**Figure 6:**
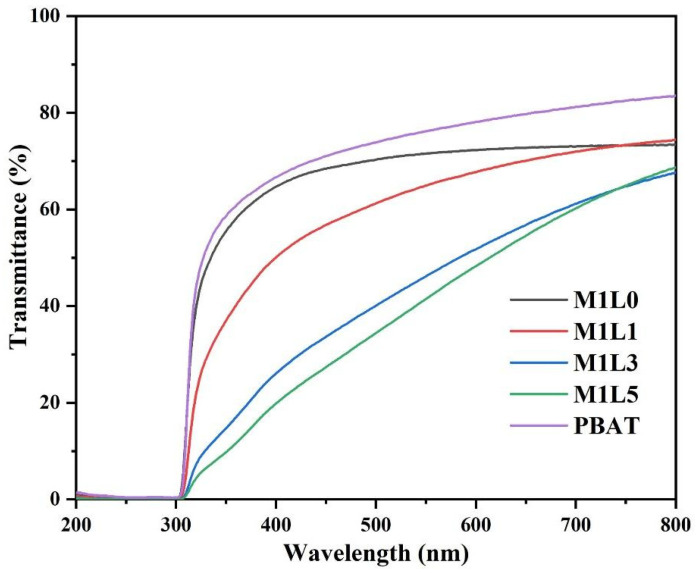
UV-VIS spectra of starch films with different lignin contents.

Ultraviolet rays were the main factor causing the rapid aging of the mulch film. Strengthening the anti–ultraviolet properties of the film can effectively extend the service life of the film. The results of ultraviolet-visible light spectra of starch-based films with different lignin content were shown in Figure 6. We found that the light transmittance of pure PBAT film in the visible light region (400–760 nm) is not very high, which may be attributed to the semi-crystalline structure of PBAT. With the addition of starch, the light transmittance of the film decreased slightly, which was not much different from PBAT. This was mainly because the modified starch was evenly dispersed in the PBAT, and light spreads evenly in the film due to uniformity. With the addition of lignin, the light transmittance of the visible light region of the film gradually decreased as the content increased. However, the barrier properties of lignin to ultraviolet light were very strong. It can be seen from the figure that pure PBAT film and starch-based film can shield more than 90% of UV-B ultraviolet light (280–315 nm). However, the film without the lignin group had almost no barrier performance in the ultraviolet region (UV-A, 315–400 nm), and the transmittance was about 60%. The transmittance of the 5% lignin film in the ultraviolet region (UV-A) was about 15%, and the visible lignin had a very strong ability to absorb ultraviolet light. This was mainly due to the large number of aromatic structures contained in lignin, among which the *P*-π conjugation effect of the phenylpropane structure could efficiently absorb a large amount of ultraviolet light [17,27]. In addition, the above results showed that the incorporation of lignin into starch-based films could significantly improve the UV barrier properties and had potential application value in the fields of food packaging bags and agricultural films.

### 3.7. UV Aging Performance Analysis

**Figure 7:**
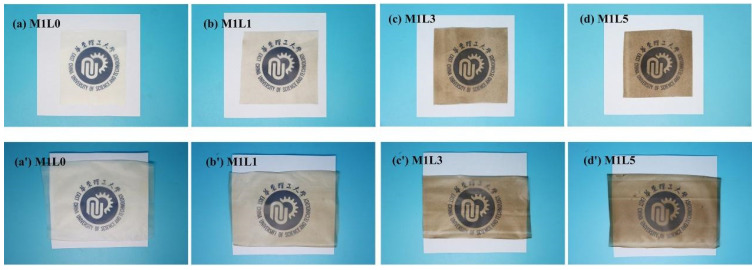
Photos of starch/PBAT composite film before and after UV aging. Before: (**a**) M1L0, (**b**) M1L1, (**c**) M1L3, (**d**) M1L5; After: (**a’**) M1L0, (**b’**) M1L1, (**c’**) M1L3, (**d’**) M1L5.

The photographs of the starch-based film before aging (a–d) and after UV aging (a’–d’) were shown in Figure 7. It could be seen from Figure 7a–d that the film without lignin was light yellow and translucent and had good light transmittance. The addition of lignin made the film brown. As the content of lignin increased, the brown color of the starch-based film gradually deepened and the light transmittance decreased. However, the M1L5 film could still see the logo on the bottom through the film. It showed that the lignin film still has a certain light transmittance. In addition, through the macro photos, it could be roughly seen that the lignin is more uniformly dispersed in the system, and there was no agglomeration phenomenon. In the system, after adding lignin, it could still be prepared by blow molding, indicating that lignin and starch-based composites have good compatibility and there is no phase separation phenomenon. It could be seen from Figure 7 that the color of the MIL0 film after UV aging (Figure 7a’) is obviously yellower than that when it is not aging (Figure 7a). The color of the lignin–containing film was still dark brown, and the change was not obvious. Combining the ultraviolet light transmission, it could be basically determined that lignin had a significant effect on resisting ultraviolet aging. That is, the starch-based film doped with lignin also has the function of anti-ultraviolet aging.

In order to further explore the resistance of lignin to ultraviolet light, four groups of starch-based films were placed in an ultraviolet aging box for continuous aging for 100 h. The mechanical properties of starch-based films with different lignin content (longitudinal and transverse) after UV aging were shown in Table 4. The results clearly showed that 100 h of continuous UV irradiation, the M1L0 film was obviously aged, and its performance decreased. However, the addition of lignin can effectively alleviate this problem [27]. With the increase of lignin in the film, the aging of the film was significantly reduced. The longitudinal CRσ and CRε of the M1L5 film were 85.43% and 80.82%, respectively, which were significantly higher than that of the M1L0 film. The benzene ring and conjugated quinone structure of lignin provided good resistance to ultraviolet aging [28]. The higher the lignin content, the smaller the performance degradation. The visible lignin content was directly proportional to the absorption of ultraviolet light. The national standard GB/T 35795-2017 for biodegradable agricultural mulch film stipulated that the elongation at break of agricultural mulch film with a service life of more than 120 days after UV aging for 100 h must reach 120% in the longitudinal direction and 200% in the transverse direction. Obviously, all four groups of starch-based films could meet the requirements.

### 3.8. Sealing Strength

**Figure 8:**
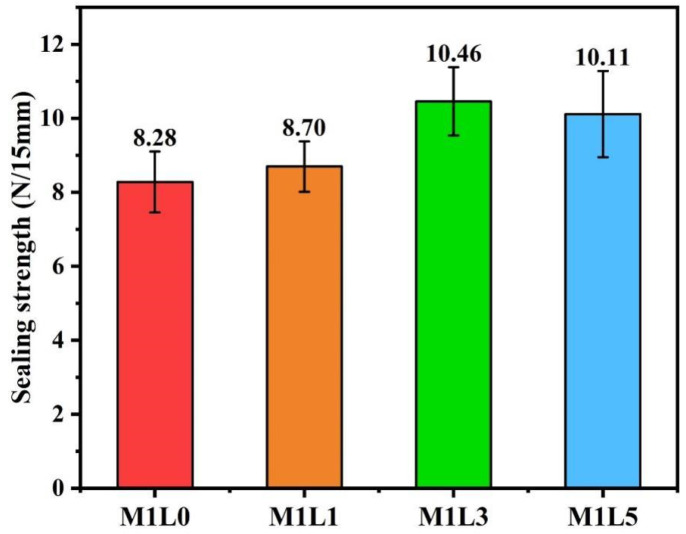
Heat-sealing performance of starch/PBAT films with different Lignin contents.

The heat–seal performance of the anti-ultraviolet aging starch/PBAT film doped with different lignin ratios was shown in Figure 8. The results showed that the addition of lignin slightly improved the heat-seal performance of starch/PBAT film, which was mainly due to the rigidity of lignin. Under the pressure and temperature provided by the heat sealer, the film becomes a molten state, and the molecular chains are intertwined and penetrated at the sealing interface to be sealed as a whole [29]. It can be seen from Figure 8. that the heat seal strength of M1L0 without lignin is 8.28 N/15mm, while the heat seal strength of M1L3 is the highest 10.46 N/15 mm. The results show that the doping of lignin is beneficial to improve the heat-seal strength of starch-based films. The heat-sealing performance of the film is closely related to its interface compatibility. From the data in Table 4, the incorporation of lignin did not reduce the interfacial compatibility of starch/PBAT film but increased the heat seal strength. According to the GBT 38082-2019 standard, the sealing strength of biodegradable shopping bags weighing more than 10 kg must exceed 8 N/15mm. Obviously, the four sets of starch-based films prepared in this study completely meet the requirements.

## 4. Conclusions

Starch/PBAT films with high ultraviolet aging resistance and excellent mechanical properties were successfully continuously blown by introducing lignin with polyurethane prepolymer (PUP) as a starch modifier and physical compatibilizer and 4,4′-methylene diphenyl diisocyanate (MDI) as crosslinker. Lignin was uniformly dispersed in the system and had little effect on crystallization performance and compatibility. PUP modifying starch and MDI crosslinking between modified starch and PBAT endowed the composite material with excellent processing ability, good mechanical properties and high starch content. The anti-ultraviolet aging performance improved with the increased lignin content of the starch/PBAT films. The starch-based film with a lignin content of 1wt.% had the best mechanical properties with longitudinal tensile strength of 15.87 MPa and an elongation at break of 602.21%. Lignin is beneficial to improve the heat-seal strength of starch-based films. The heat seal strength of M1L3 is the highest at 10.46 N/15mm. The anti-ultraviolet aging starch-based films are expected to be used as agricultural mulch films, nursery mulch films, weeding films, vegetable greenhouse films, and pesticide sustained-release films.

## Figures and Tables

**Table 1:** Starch-based films with different mixing ratios.

Samples	Modified Starch (wt.%)	PBAT (wt.%)	MDI (phr)	Lignin (phr)
M1L0	50	50	1	0
M1L1	50	50	1	1
M1L3	50	50	1	3
M1L5	50	50	1	5

Phr: The number of (MDI or Lignin) added per 100 parts of modified starch and PBAT by mass.

**Table 2:** Contact angle and WVTR of starch/PBAT films with different lignin contents.

Samples	Contact Angle (°)	WVTR (g/m^2^/day)
Blank	–	6828.95 ± 88.62
M1L0	80.00 ± 0.50	865.57 ± 14.02
M1L1	78.75 ± 0.25	907.04 ± 13.12
M1L3	77.00 ± 0.50	1125.18 ± 12.74
M1L5	74.25 ± 0.25	1185.00 ± 9.92

**Table 3:** Tensile properties of starch/PBAT films with different lignin contents.

Samples	Direction	Tensile Strength (MPa)	Elongation at Break (%)
**M1L0**	Horizontal	14.04 ± 1.29	619.03 ± 28.54
	Longitudinal	14.23 ± 1.77	596.41 ± 22.68
**M1L1**	Horizontal	15.31 ± 0.98	715.88 ± 42.52
	Longitudinal	15.87 ± 1.58	602.21 ± 39.17
**M1L3**	Horizontal	14.58 ± 1.53	658.96 ± 29.98
	Longitudinal	15.11 ± 1.36	562.47 ± 33.86
**M1L5**	Horizontal	14.23 ± 0.98	671.36 ± 38.12
	Longitudinal	15.03 ± 1.33	588.41 ± 34.72

**Table 4:** The tensile properties of starch/PBAT films before and after UV-aging disposition.

Samples	Tensile Strength (MPa)			Elongation at Break (%)		
	Before	After	CR_σ_	Before	After	CR_ε_
M1L0-L	14.23 ± 1.77	10.51 ± 1.52	76.25%	596.41 ± 22.68	421.87 ± 21.76	70.73%
M1L0-H	14.04 ± 1.29	9.96 ± 1.03	70.94%	619.03 ± 28.54	425.51 ± 25.32	68.74%
M1L1-L	15.87 ± 1.58	12.83 ± 0.89	80.84%	602.21 ± 39.17	458.65 ± 31.25	76.16%
M1L1-H	15.31 ± 0.98	11.63 ± 0.68	75.96%	715.88 ± 42.52	521.59 ± 30.26	72.85%
M1L3-L	15.11 ± 1.36	12.46 ± 1.31	82.46%	562.47 ± 33.86	443.72 ± 26.37	78.89%
M1L3-H	14.58 ± 1.53	11.37 ± 0.78	77.98%	658.96 ± 29.98	495.22 ± 35.68	75.15%
M1L5-L	15.03 ± 1.33	12.84 ± 1.16	85.43%	588.41 ± 34.72	475.61 ± 36.41	80.82%
M1L5-H	14.23 ± 0.98	11.52 ± 1.32	80.95%	671.36 ± 38.12	517.94 ± 30.84	77.14%

## Data Availability

The author declares that all the data in the article are true and valid. If you need to quote, please indicate the source.
